# Complete resolution of rectal cancer with liver metastases after palliative chemoradiotherapy and unplanned surgical resection—a case report

**DOI:** 10.1093/jscr/rjad060

**Published:** 2023-02-22

**Authors:** Zhi S Choong, King H Wong, Tze W W Yang, Margaret Nguyen, Zeev Duieb

**Affiliations:** General Surgery, Albury Wodonga Health, Albury, NSW, Australia; General Surgery, West Gippsland Healthcare Group, Warragul, VIC, Australia; General Surgery, Albury Wodonga Health, Albury, NSW, Australia; Radiology, Albury Wodonga Health, Albury, NSW, Australia; General Surgery, Monash Health, Clayton, VIC, Australia

## Abstract

Metastatic colorectal cancer has poor prognosis for many patients at time of diagnosis with <20% 5-year survival rate. Recent advancements in palliative chemotherapy have improved patient outcomes as median survival has increased almost 2-fold. We report a 44-year-old gentleman who initially underwent palliative chemoradiotherapy and subsequently a Hartmann’s procedure for ypT3N1M1 upper rectal adenocarcinoma with multiple liver metastases. Fortuitously, he made a remarkable recovery with complete radiological resolution of liver metastasis post-operatively. The patient has remained in remission for the past 10 years.

## INTRODUCTION

Patients with metastatic colorectal cancer (CRC) often present with poor prognosis at time of diagnosis. Palliative chemotherapy, 5-fluorouracil (5-FU) with leucovorin and oxaliplatin (FOLFOX) in particular, remains the gold standard treatment of choice for unresectable metastatic CRC. Surgical interventions to remove the primary tumour in advanced disease are limited and currently only warranted in the event of obstruction or bleeding. This case would hopefully add value to the literature for clinicians to consider the role of surgical resection after palliative chemoradiotherapy in patients with advanced CRC and liver metastasis (CRCLM) in the future.

## CASE REPORT

A 44-year-old Mr TM presented in October 2011 with a 2-week history of rectal (PR) bleeding, pencil-like bowel motions and right upper quadrant pain. Colonoscopy revealed a non-obstructing poorly differentiated upper rectum adenocarcinoma, 10 cm from the anal verge. Completion staging computerised tomography (CT) scan demonstrated multiple liver metastases in Segments 2, 4A, 5–8, maximally 94 mm in diameter as shown in Table 1.

**Table 1:** Comparison of the dimensions of target lesions of the patient on CT scan

**Dimensions of target lesions (mm)**				
**Location date**	**Baseline scan 26 October 2011**	**Pre-op scan 25 September 2012**	**Follow-up scan 15 January 2013**	**Follow-up scan 12 March 2013**
Liver segments 7/8 high	54	13	9	Not detected
Liver segments 2/4A	94	21	16	11
Liver segments 7/8 low	69	25	12	11
Liver segments 5/6	72	13	4	5
Liver segment 6	40	14	10	11
Rectal primary	35	20	Resected	Resected

He was deemed suitable for palliative chemoradiotherapy and underwent 13 cycles of FOLFOX and 1 cycle of SIRTEX, eventually ceased because of severe peripheral neuropathy. He responded positively to treatment, with more reduction in the size of both his primary rectal cancer and liver metastasis on serial CT scans.

Unfortunately, in August 2012, 6 months after the completion of palliative chemoradiotherapy, the patient began to develop worsening PR bleeding. An urgent colonoscopy demonstrated a slight increase in size of the rectal tumour from 20 to 30 mm, with evidence of ulceration, which was likely the cause of his symptoms.

Following discussion within a multidisciplinary team, the patient underwent an uncomplicated palliative open Hartmann’s procedure in December 2012 for ypT3N1M1 upper rectal adenocarcinoma (1 out of 14 involved lymph nodes, immunoperoxidase staining of the tumour was negative for CK7, CK28 and CD18). Post-operatively, the patient opted for no further chemoradiotherapy and was also not deemed for surgical intervention regarding the liver metastases.

**Figure 1:**
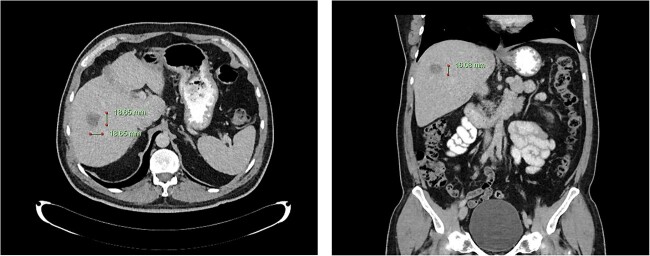
Computed tomography dated 25 September 2012 of the abdomen and pelvis measuring 18.65 mm (anterior–posterior) lesion at liver segment 5/6 in axial (left) and coronal (right) view.

**Figure 2:**
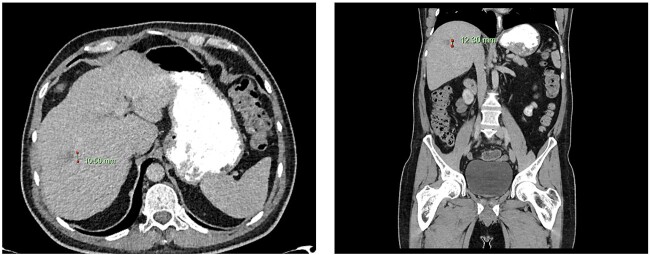
Computed tomography dated 15 January 2013 of the abdomen and pelvis measuring 10.50 mm (anterior–posterior) lesion at liver segment 5/6 in axial (left) and coronal (right) view.

**Figure 3:**
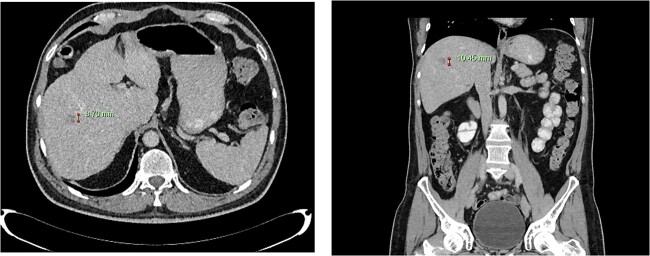
Computed tomography dated 12 March 2013 of the abdomen and pelvis measuring 8.70 mm (anterior–posterior) lesion at liver segment 5/6 in axial (left) and coronal (right) view.

**Figure 4:**
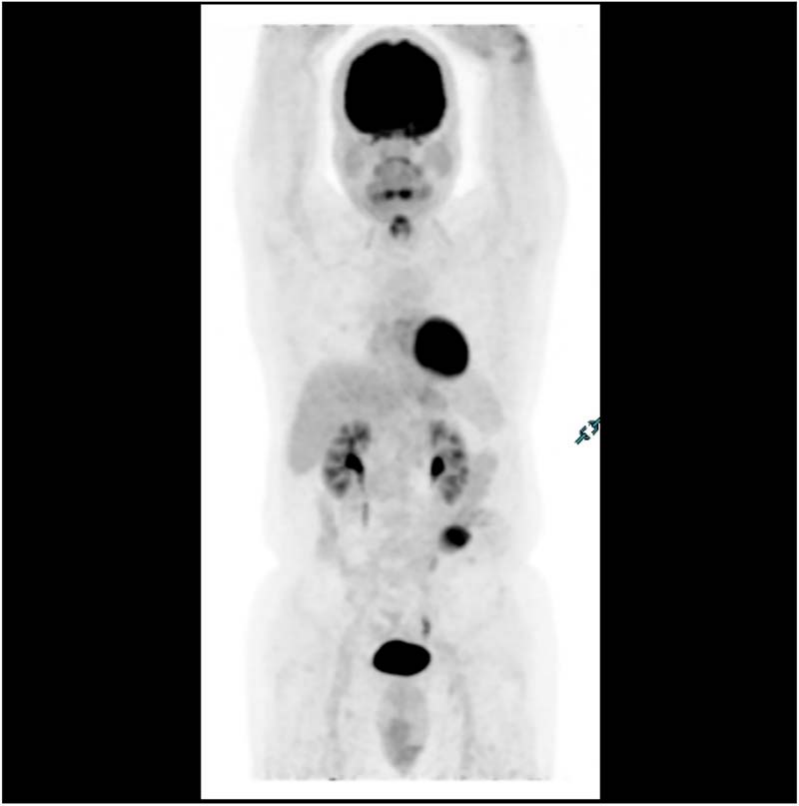
PET scan in 2018 demonstrating no evidence of FDG avid regional nodal or distant metastasis, especially in the liver.

Subsequent surveillance imaging including magnetic resonance imaging and CT scans demonstrated gradual reduction and eventual complete resolution of the liver metastases (refer to Figs 1–4). Furthermore, routine colonoscopy and a positron emission tomography (PET) scan revealed no evidence of locoregional cancer recurrence nor distant metastatic disease present; essentially rendering the patient cured from an initial diagnosis of metastatic rectal cancer. It has been at least a decade since his initial diagnosis and the patient remains in remission.

## DISCUSSION

CRC is the third most diagnosed cancer in Australia [1], with patients most commonly presenting with symptoms including rectal bleeding, abdominal pain or anaemia [2]. The presence of symptoms upon diagnosis is associated with more advanced disease, compared with cases diagnosed incidentally or via screening [2]. In all, 18% of diagnoses in Australia are clinically Stage IV CRC with poor prognosis of <20% 5-year survival rate [1]. Recent advancements in chemotherapy development have improved patient outcomes as median survival has increased from 1 year to 30 months [3]. FOLFOX remains the gold standard chemotherapy for unresectable metastatic CRC. In a particular study, complete disappearance of all clinically assessable disease was found in 5% of patients post-FOLFOX, where good performance status and patients with liver-only metastasis were identified as the two independent prognostic factors. Additionally, 22% of patients with a mean number of 12 FOLFOX cycles were able to undergo further surgical metastasis removal [4].

Moreover, in incurable disease, the incorporation of palliative surgery with chemotherapy significantly improves quality of life and symptom management [5]. Surgical resection with curative intent is the treatment of choice for patients who present with non-metastatic CRC [6]. Patients with limited metastases, such as isolated hepatic or pulmonary metastasis, may benefit from regional surgical treatment in combination with systemic chemotherapy [7]. In patients with advanced metastatic colorectal disease, surgical interventions are limited and only warranted in the event of obstruction or bleeding. In particular, Hartmann’s procedure and abdominoperineal resection are both considered appropriate and symptom-alleviating surgeries for palliation in patients with advanced rectal cancer [5, 8]. Endoscopic stenting is another option, however is associated with shorter patency duration, shorter median survival rate and higher late adverse events such as tumour ingrowth and stent perforation as compared with palliative surgery [9].

Understandably, there are limited guidelines for surveillance of resected Stage IV CRC as poor prognoses are expected in most patients. For patients with CRCLM, liver resection is the definitive treatment offering the best chance at long-term survival [10]. However, liver resection is not commonly performed in CRCLM patients, with a study reporting only 6.1% of patients undergoing liver resection because of old age and multiple comorbidities and hence perceived higher morbidity, with a subsequent 5-year survival rate of 30% [11].

Although cases of the radiological ‘disappearing metastasis’ phenomenon post-chemotherapy for CRCLM have previously been reported [10, 12], we believe this is the first reported case of a patient with complete radiological resolution of rectal cancer with liver metastases after palliative chemoradiotherapy and unplanned surgical resection. This case would hopefully add value to the literature for future studies to consider the role of surgical resection after palliative chemoradiotherapy in patients with advanced CRCLM.

## CONFLICT OF INTEREST STATEMENT

None declared.

## FUNDING

None.
